# Fighting typhoid fever in Tanzania: the importance of evidence for effective prevention and control amid urgency to act

**DOI:** 10.1097/MS9.0000000000003440

**Published:** 2025-05-30

**Authors:** Christian Tague, Amidu Alhassan, Maher Ali Rusho, Saim Mahmood Khan, Soham Bandyopadhyay, Innocent H. Peter Uggh, Faizullah Jafar, Gbènonminvo Enoch Cakpo, Farheen Naaz, Elie Kihanduka, Samson Hangi, Excellent Rugendabanga, Aymar Akilimali

**Affiliations:** a Department of Research, Medical Research Circle (MedReC), Bukavu, DR Congo; b College of Health And Allied Sciences, University of Cape Coast, Cape Coast, Ghana; c Department of Medical Biophysics, University of Toronto, Toronto, Canada; d Faculty of medicine, Karachi Medical and Dental College, Karachi Pakistan; e Clinical Neurosciences, Clinical and Experimental Sciences, Faculty of Medicine, University of Southampton, Southampton, Hampshire, United Kingdom; f Oxford University Global Surgery Group, Nuffield Department of Surgical Sciences, University of Oxford, Oxford, United Kingdom; g Kilimanjaro Clinical Research Institute, Moshi, Tanzania; h Department of Biotechnology, School of Chemical and Life Sciences, Jamia, Hamdard, New Delhi, India; i Higher Institute of Population Sciences, Joseph KI-ZERBO University, Ouagadougou, Burkina Faso; j Medical college, Deccan College of Medical Sciences, Hyderabad, India; k International Veterinary Vaccinology Network, The Roslin Institute University of Edinburgh, Edinburgh, United Kingdom; l Global Schistosomiasis Alliance, London, United Kingdom

*Dear Editor,*

Typhoid fever (TF) is caused by the bacterium *Salmonella enterica* serovar Typhi (*S. typhi*). The typical clinical picture usually involves high fever, weakness, and abdominal pains alongside other symptoms. Mainly, transmission is through ingestion of contaminated food and water. The disease remains common because of poor sanitation, inadequate clean water, and an underdeveloped healthcare system, despite improvements in medical science^[1,2]^.

TF is a major public health problem in most developing countries. According to the World Health Organization (WHO), in 2019, an estimated 9 million people contracted TF and 110 000 people died from it that year^[3]^. TF is endemic in Tanzania with more than 79 000 cases per year – approximately 140 cases per 100 000 population – ^[4]^ and nearly 2000 deaths. According to recent health reports, the incidence of TF in Tanzania is particularly high in areas where sanitation and water quality are poor^[5]^. TF causes a substantial burden to individuals, families, and the community in Tanzania. The high incidence of TF in children and young adults in Tanzania leads to many lost productive years, as well as greater financial insecurity for affected families^[6]^. During outbreaks, healthcare facilities become overburdened and fail to provide timely or adequate treatment to all. Serious long-term consequences of TF are the development of chronic carriers of *S. typhi* and complications like intestinal perforation, which require surgical treatment and may be fatal if not treated^[7]^. As such, the putative burden of TF in Tanzania calls for urgent and comprehensive strategies to combat this public health threat. Our article contributes to the literature by highlighting the evidence-based interventions in preventing and controlling TF in Tanzania. TF control and prevention require a multi-combinational approach that includes the access of water, sanitation and hygiene infrastructure improvement, vaccination campaigns, public health education, and maintenance of sound surveillance systems for the disease.

Vaccination is a critical intervention for both short- and long-term control of TF. Two vaccines are widely available: Ty21a and Vi polysaccharide (ViP). The Ty21a vaccine refers to the live-attenuated oral typhoid vaccine, while VIP is an injectable vaccine. Both are used to prevent TF, but they differ in administration, efficacy, and target age groups. The Ty21a vaccine is typically given as three oral doses and provides about 50% protection, while the ViP vaccine is administered as a single injection and offers about 69% protection in the first year, with cumulative efficacy of 55% by the third year. Ty21a prevents around half of typhoid cases during the first three years after vaccination, and the duration of protection can last for up to 7 years. Ty21a is not recommended for individuals with weakened immune systems, and children under 5 are not typically vaccinated with Ty21a. ViP is suitable for children as young as 2 years old, but may have a shorter duration of protection. Newer typhoid conjugate vaccines (TCVs) are increasingly seen as the preferred option over ViP and Ty21a vaccines, particularly in younger children. TCVs are preferred due to their improved immunological properties, wider applicability (including infants), expected longer duration of protection, and ability to induce a booster response^[8]^. The WHO endorses the use of TCVs because they are effective, safe, and are indicated in children. The WHO has recently recommended the Vi tetanus toxoid conjugate vaccine as the preferred vaccine for all ages^[9]^. Common side effects of vaccines include pain, mild fever, redness at the injection site, or gastrointestinal discomfort in the case of oral vaccines. Severe adverse reactions are rare^[10]^. Spreading the awareness about availability of TCVs, and assuring the public about the safety and efficacy of TCVs is necessary for vaccination programs to be an effective measure for the control of TF.

Public health education can also empower communities to improve hygiene, improves surveillance, and allows for earlier recognition of symptoms for earlier treatment. Hygiene practices – such as handwashing with soap after using the toilet or before eating – can be taught through demonstrations in public health campaigns. Additionally, instructing people on safe water and food-handling practices, such as drinking boiled water and avoiding raw vegetables that may be contaminated with fecal matter, is important. Availability of clean water and sanitation in many parts of the world has remained a crucial factor in breaking the transmission cycle of *S. typhi*^[5]^.

Engaging local leaders and schools in disseminating this information is crucial. This strategy is cost-effective and can be utilized within community outreach programs, which ensures widespread awareness and behavioral change, maximizing the impact of these educational efforts.

Investment into infrastructure to provide clean water and good sanitation facilities in socially disadvantaged areas will greatly reduce the transmission of *S. typhi*. The involvement of local stakeholders in the planning and implementation stages through community-based approaches enhances the sustainability and effectiveness of the projects, as the maintenance of the implemented projects are improved while ensuring continued access to safe water and sanitation.

In addition, there is a need for enhanced surveillance systems that would permit timely monitoring and response to the occurrence of TF. A well-developed surveillance system can detect an outbreak early and enable quick response measures to prevent its spread.

TF presents a significant public health challenge in Tanzania. This has required evidence-based, urgent intervention in the prevention and control of the disease. Through improved water, sanitation, and hygiene infrastructures; vaccination programs; improved surveillance systems; and more public health education, Tanzania can make significant steps toward a reduced burden of TF. This involves stakeholders at the government, nongovernmental, and community levels for sustainable enhancement of public health to mitigate the threat of TF.

## Data Availability

Not applicable.
